# Cardiac Metastasis in a Patient with Head and Neck Cancer: A Case Report and Review of the Literature

**DOI:** 10.1155/2019/9581259

**Published:** 2019-04-18

**Authors:** Joseph K. Kim, Kunal Sindhu, Richard L. Bakst

**Affiliations:** ^1^SUNY Downstate Medical Center, Department of Radiation Oncology, Brooklyn, New York, USA; ^2^Mount Sinai Medical Center, Department of Radiation Oncology, One Gustave L. Levy Place, Box 1236, New York, New York, USA

## Abstract

Cardiac metastasis from a primary head and neck cancer is a rare finding. Most patients with cardiac metastases have nonspecific symptoms that may vary depending on the severity and location of the lesion. Due to the infrequency of reported cases, there are no clear guidelines for the diagnosis or management of cardiac metastasis in head and neck cancer patients. In this report, we discuss the case of a patient with a primary diagnosis of oral tongue cancer who developed a cardiac metastasis that was detected antemortem.

## 1. Introduction

Cardiac metastases in patients with head and neck squamous cell carcinoma (HNSCC) are a very rare finding [1]. Most cases of cardiac metastasis are clinically silent with a majority of cases detected in the postmortem setting. If cardiac metastasis is diagnosed in a living patient, the clinical presentation is often variable with nonspecific symptoms. Here, we discuss the clinical presentation, diagnosis, and management of a patient with oral cavity cancer who developed a metastasis to the heart.

## 2. Case Presentation

**Figure 1:**
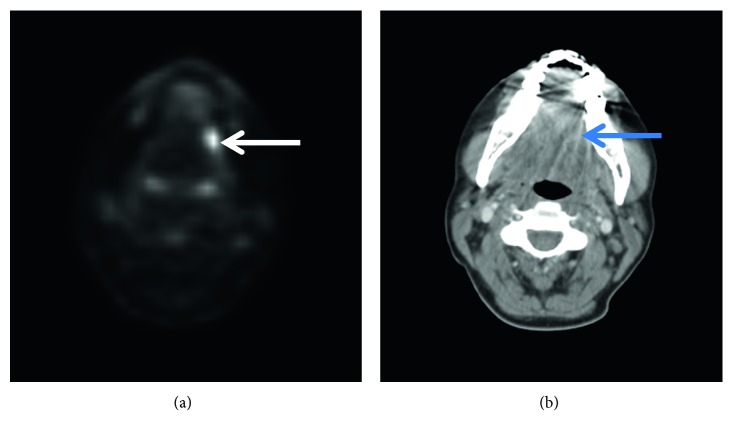
Initial axial PET scan (a) and CT scan (b) demonstrating hyperactivity along the left lateral tongue ((a) white arrow; (b) blue arrow).

A 46-year-old white female with a 15 pack-year smoking history initially presented 3 years ago with intense pain in the left side of the mouth that radiated to her left ear for 2 months. She was found to have a 2 cm exophytic lesion on the left lateral border of her tongue that was diffusely keratotic and extremely tender on examination. Fiberoptic laryngoscopy revealed normal findings in the nasopharynx, oropharynx, and hypopharynx. Histological biopsy of the tongue lesion confirmed well-differentiated squamous cell carcinoma of the left lateral border of the tongue. Radiographical findings on positron emission tomography (PET) scan showed hyperactivity along the left lateral aspect of the tongue and a mildly hypermetabolic left level IIa cervical lymph node with no evidence of distant metastases (Figure 1).

The patient was treated with a left hemiglossectomy and bilateral neck dissection. Pathologic evaluation revealed a 2.4 cm moderately differentiated, infiltrating squamous cell carcinoma of the left lateral tongue lesion invading into the skeletal muscle with a 0.5 cm maximal thickness. There was perineural invasion, but no lymphovascular invasion, and all margins were free of cancer. A total of 3 out of 22 lymph nodes were positive for carcinoma: 0 out of 10 in right neck level II-III, 2 out of 5 left level I with no extracapsular extension (ECE), 0 out of 1 left level II, and 1 out of 6 left level III with no ECE.

She was staged as pT2 pN2b M0 (stage IVA, AJCC 7th edition 2010) squamous cell carcinoma of the left lateral tongue. She received adjuvant treatment with concurrent afatinib and radiotherapy to a total dose of 6000 cGy in 30 fractions over 6 weeks to the oral cavity and bilateral necks, which was completed in 3 months after diagnosis.

Interval radiographical imaging did not show any evidence of disease recurrence or distant metastases until 2018. At that time, the patient had developed increasing left arm pain, left ear pain, and left throat pain. She also reported intermittent chest pressure, exertional dyspnea, and intermittent dizziness with positional changes.

**Figure 2:**
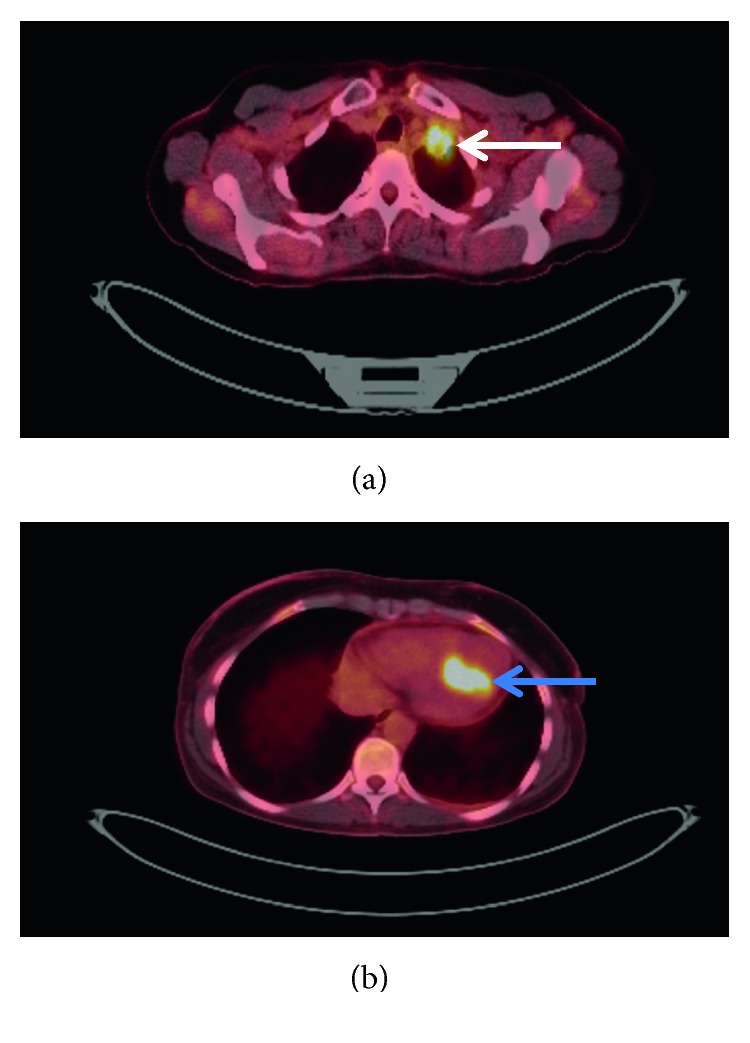
Axial PET scans revealing a new left apical pleural mass (white arrow) (a) and cardiac metastasis (blue arrow) (b).

**Figure 3:**
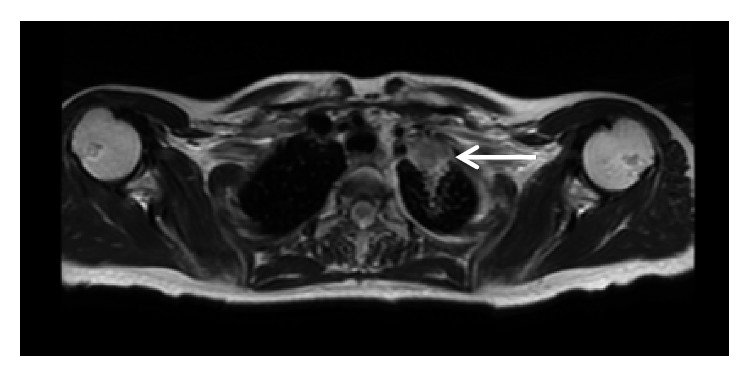
MRI of the chest demonstrating a new left apical pleural mass (white arrow).

In early 2018, a PET scan (Figure 2) and magnetic resonance imaging (MRI) of the chest (Figure 3) showed a new 3.4 cm left apical pleural mass encasing the left subclavian artery and abutting the left subclavian vein, both of which were patent. A computed tomography-guided fine-needle aspiration (CT-FNA) of the apical lung mass revealed squamous cell carcinoma.

**Figure 4:**
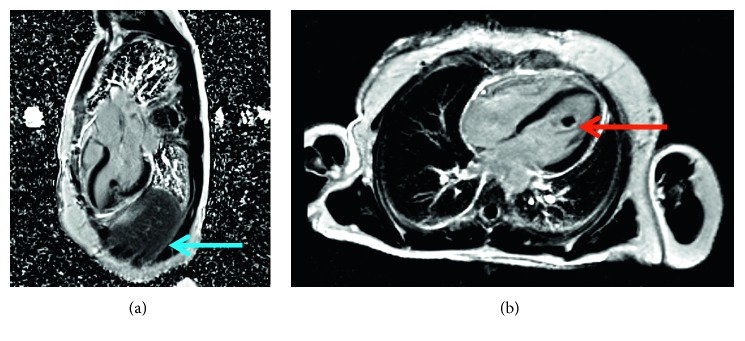
Cardiac MRI demonstrating a new mass infiltrating the left ventricular inferior myocardium, epicardial fat, and pericardium (blue arrow and red arrow).

Shortly after, the patient began systemic therapy with cisplatin and etoposide and radiation therapy to the left apical lung lesion. Following the 20th fraction of radiation therapy, a repeat computed tomography (CT) scan of the chest revealed a new 1.2 cm lesion in the inferior interventricular septum of the heart. A cardiac ultrasound was performed and demonstrated a mass in the left ventricle. The patient went on to complete radiation therapy to a total of 6000 cGy in 30 fractions. Further diagnostic imaging with a cardiac MRI was performed, which revealed a mass infiltrating the left ventricle, inferior myocardium, epicardial fat, and pericardium with associated mobile thrombus formation (Figure 4). A PET/CT scan demonstrated hypermetabolic lesions in the left neck, right thigh muscles, lung parenchyma, heart, anterior mediastinum, left scapula, and posterior right rib (Figure 2). Transthoracic echocardiogram (TTE) showed a 1.6 × 1.4 cm mobile mass in the left ventricle cavity that appeared to be attached to the base of the papillary muscle and a normal left ventricular ejection fraction of 60%. An electrocardiogram (ECG) revealed normal sinus rhythm with T-wave inversion in the inferior leads and V_3_–V_6_.

The patient initiated anticoagulation and systemic therapy with nivolumab.

## 3. Discussion

Cardiac metastases are a rare finding. Since patients are often clinically silent or have nonspecific symptoms, metastases to the heart are difficult to diagnose and usually detected in the postmortem setting during an autopsy. When patients present with symptoms, they can have highly variable clinical manifestations, including heart failure, arrhythmias, valvular disease, and cardiac tamponade. The most common primary cancers in patients with cardiac metastases include melanoma, mediastinal tumors, lung cancer, breast cancer, and leukemia [2]. In a large study of postmortem patients with a known malignancy, Bussani et al. reported a 9.1% overall incidence of cardiac metastases with only 5.3% (4 out of 75) secondary to oral cavity cancer compared to 48.4% secondary to mesothelioma, 27.8% to melanoma, and 21.0% to lung adenocarcinoma [2]. Four different mechanisms have been postulated by which cancer spreads to the heart, including direct extension, hematologic spread, lymphatic spread, and intracavitary diffusion via the inferior vena cava or pulmonary veins [2–4].

In patients with oral cavity cancer, distant metastases are seen in 4.2–23.8% of patients, with the lung, bone, and liver as the most commonly involved sites [5]. However, metastatic disease to the heart is highly unusual in patients with oral cavity cancer. The patient in this case report had an especially long duration between primary diagnosis and cardiac metastasis at almost 3 years. In a review of the literature, we found 24 cases of patients with head and neck cancer with cardiac metastases that were detected in the antemortem setting.

Due to the wide range of clinical presentations in patients with cardiac metastases, detection is often incidental. The routine use of imaging is not generally recommended in patients with head and neck cancer to detect metastatic disease unless prompted by clinical signs and symptoms or if the patient is not amenable to clinical examination. Echocardiography is the most frequent noninvasive imaging modality used to evaluate the heart. In our present literature review, echocardiography was the initial imaging modality in more than 50% of the cases of cardiac metastasis from a primary head and neck cancer (Table 1). Echocardiography allows for assessment of cardiac function, including valvular and ventricular competency, as well as identification of any intraventricular masses or structural wall abnormalities [6]. The diagnostic accuracy of echocardiography has been reported to be as high as 80%, which makes it a good initial method to evaluate suspected cardiac tumors [7]. Other imaging modalities such as CT and MRI may provide a more comprehensive assessment with additional detail, including evaluation of the pericardium and extracardiac disease [8]. Cardiac MRI offers the advantages of excellent contrast resolution and distinction of tumor from the myocardium or thrombus in comparison with CT or ultrasound [9]. MRI also allows for simultaneous assessment of the surrounding structures, including the mediastinum, lungs, and pleura [6]. Particularly in patients with primary tumor of the head and neck, PET/CT may provide additional utility in detecting metastatic disease involving the heart. In a long-term outcome study of PET/CT imaging in head and neck cancer patients treated with definitive or adjuvant radiation therapy, PET/CT had a 99% negative predictive value in the assessment of the primary site and neck, and negative findings were associated with significantly improved disease-free survival and overall survival. However, there was a high rate of false-positive results at the primary site, with a positive predictive value of 32.1% [10]. Therefore, the use of PET/CT at 3 months post-RT is generally practiced. Current National Comprehensive Cancer Network (NCCN) guidelines do not recommend the use of routine follow-up imaging unless clinically indicated and no evidence to demonstrate a benefit of surveillance imaging after 6 months [11]. However, multiple case reports have reported that the use of PET/CT may lead to improved accuracy and earlier detection of cardiac metastases in patients with head and neck cancer [4, 12–15]. Some studies have suggested that routine ECG may have a diagnostic value, but findings are often nonspecific, and further confirmation with imaging would be required [15–17]. Table 1 provides a literature review of patients with an antemortem diagnosis of cardiac metastasis from a primary head and neck cancer. In our present study, the patient had already developed metastatic disease in the lung prior to detection of the cardiac metastasis, which was incidentally detected on a review staging CT scan.

There is no standard of care in the treatment of cardiac metastases. Many patients are not surgical candidates due to the location of disease and are treated with palliative chemotherapy and supportive care, but prognosis is often poor. In our present study, the patient initially had presented with metastatic disease to the lung and began treatment with cisplatin-based chemotherapy and radiotherapy, but developed progression of disease and a new cardiac metastasis. In the current era, novel immunotherapeutic drugs, such as pembrolizumab or nivolumab (anti-PDL-1 antibodies), may have a significant impact on the survival outcomes of these patients [27–29]. Upon detection of the cardiac metastasis, the patient began treatment with nivolumab, which is approved for patients with recurrent and metastatic head and neck cancer that is refractory to cisplatin chemotherapy.

In conclusion, metastatic disease to the heart in oral cavity cancer is an uncommon finding, lacking optimal guidelines in terms of diagnosis and management. Diagnosis is quite challenging since cardiac metastases are often clinically silent, and there is no clear beneficial role of routine surveillance imaging. In symptomatic patients, a multimodality approach using imaging such as PET/CT, cardiac MRI, echocardiogram, and ECG findings should be used to confirm the location and extent of disease, which may help to guide treatment options.

## Figures and Tables

**Table 1:** Literature review of cardiac metastasis in head and neck cancer detected ante-mortem.

Patient	Study	Year of publication	Primary site	Primary treatment	Location of metastasis	Signs/symptoms	ECG findings	Biopsy proven cardiac metastasis	Treatment for cardiac metastasis	Initial imaging modality to detect cardiac metastasis	PET scan used?
1	Werbel et al. [18]	1985	Base of tongue	Hemiglossectomy	Mediastinal mass compressing right ventricular outflow tract and encasing the ascending aorta, right atrium mass	Intermittent positional chest pain, dysphagia, weight loss	New ST depression with T-wave inversions anteriorly	Yes	Surgical exploration via modified right-sided Chamberlain procedure, but deemed unresectable. Planned to proceed with radiotherapy, but patient expired before initiation	2D Echocardiogram	No

2	Rivkin et al. [19]	1999	Right base of tongue	Local excision and adjuvant radiotherapy to primary site and bilateral neck	Right ventricle	Chest pain, lower extremity edema	Mild ST elevation in V4 and V5, atrial fibrillation with ST elevation in V2 to V6 and Q waves in V2 and III	Yes	Chemotherapy with cisplatin, 5-FU, bleomycin, and methotrexate	Chest X-ray and Echocardiogram	No

3	Schwender et al. [20]	2002	Right buccal mucosa	Chemotherapy with cisplatin and radiotherapy	Pericardial effusion	Weakness, lightheadedness, dyspnea	Atrial fibrillation with rapid ventricular response	Yes	None	Chest X-ray, CT Chest	No

4	Zemann et al. [13]	2007	Oral cavity	Right mandibulectomy, right hemiglossectomy, right radical neck dissection, and left supraomohyoid dissection with immediate microvascular flap reconstruction followed by adjuvant radiotherapy to right lower jaw and right neck to 60 Gy/50Gy	Right ventricle	Respiratory distress	Normal findings	No	None	CT Chest	No

5	Hans et al. [21]	2008	Base of tongue	Induction chemotherapy (5-FU/cisplatin), glossectomy and left radical neck dissection and adjuvant radiotherapy to primary site and neck to 60 Gy/46 Gy	Right ventricle extending into the pulmonary infundibulum	Dyspnea, lower extremity edema, hemoptysis	Right bundle branch block	No	None	CT Chest	No

6	Tsai et al. [22]	2010	Left retromolar trigone	Segmental mandibulectomy and ipsilateral modified radical neck dissection followed by adjuvant radiotherapy to primary site and whole neck to 64 Gy	Pericardial effusion, mediastinal mass	Progressive dyspnea, tachycardia, pulsus paradoxus	Low QRS voltage	Yes	Chest tube insertion and drainage, emergency thoracotomy, palliative chemotherapy with cisplatin and cetuximab	2D Echocardiogram	Yes

7	Nagata et al. [1]	2012	Right lingual	Preoperative concurrent chemoradiation therapy to 30 Gy followed by partial glossectomy and right radical neck dissection and rectus abdominis musculocutaneous flap reconstruction followed by adjuvant chemotherapy	Left atrium to the left pulmonary vein, Pericardial effusion	Fever	—	Yes	Resection of cardiac mass	Chest CT and Echocardiogram	Yes
8			Left soft palate	Preoperative concurrent chemoradiation therapy to 40 Gy followed by partial maxillectomy and radical neck dissection followed by adjuvant chemotherapy	Right atrium and right ventricle, pericardial effusion	—	Right bundle branch block and borderline Q wave	No	None	Chest CT	Yes

9	Onwuchekwa and Banchs [23]	2012	Right oral tongue	Right partial glossectomy and extensive neck dissection	Right ventricle invading interventricular septum and left ventricle	Syncope, mild dyspnea	Sinus rhythm	No	None	CT angiogram and 2D echocardiogram	No
10			Left oral tongue	Concurrent chemoradiotherapy, left partial glossectomy, left neck dissection	Anteroseptal wall of the left ventricle extending toward the right ventricular outflow tract, pericardial effusion	Palpitations, dyspnea	Sinus rhythm with ST elevation in the anterolateral leads	No	Radiotherapy and chemotherapy	Chest X-Ray and 2D echocardiogram	No

11	Yadav et al. [16]	2014	Right piriform sinus	Chemotherapy with cisplatin and radiotherapy	Right and left ventricular apex and distal interventricular septum	None	Inferior and anterolateral ST elevation	Yes	Pemetrexed and gemcitabine	PET/CT	Yes
12			Oral tongue	Partial glossectomy	Left and right ventricle with extension to chordae tendinae	Presented with pneumonia	New anterolateral myocardial infarction (ST elevation)	No	None	Chest X-Ray and Echocardiogram	No

13	Puranik et al. [4]	2014	Left buccal mucosa	Concurrent chemoradiotherapy	Left ventricular myocardium	Weight loss	—	No	Palliative chemotherapy	PET/CT	Yes
14			Right lateral oral tongue	Wide excision and right lateral neck dissection	Left ventricular myocardium	Swelling over ala of nose	—	No	Palliative chemotherapy	PET/CT	Yes
15			Right Vallecula	Chemoradiotherapy	Right ventricular myocardium	—	—	—	Palliative chemotherapy	PET/CT	Yes

16	Pattni et al. [5]	2015	Left retromolar trigone	Began radiotherapy, but then elected to pursue surgery with curative intent-Cardiac metastasis was detected and surgery was cancelled	Apex of right ventricle extending to the tricuspid valve	Central chest “heaviness,” tachycardia, irregularly irregular pulse	ST-segment elevation	No	None	Transthoracic echocardiogram	No

17	Browning et al. [14]	2015	Base of tongue	Radiotherapy	Anterior wall of right ventricle	—	—	No	None	PET/CT	Yes

18	Martell et al. [24]	2016	Right retromolar trigone	Pharyngotomy and segmental mandibular resection, right selective neck dissection, free fibular flap reconstruction followed by adjuvant concurrent chemoradiotherapy to 60 Gy with cisplatin	Right ventricle, prevascular lymph node, pericardial effusion	Acute dyspnea, palpitations	Rapid atrial fibrillation	Yes	Planned for palliative radiotherapy to 20 Gy in 5 fractions, but patient expired prior to treatment	Echocardiogram and CT Chest	No

19	Vaduganathan et al. [25]	2016	Larynx	Unknown	Left ventricle	Arrhythmia	Stable ventricular tachycardia	No	Permanent pacemaker placement and systemic chemotherapy	Transthoracic echocardiogram	No

20	Malekzadeh et al. [3]	2017	Oral tongue	Right hemiglossectomy and adjuvant radiotherapy	Right ventricle	Acute chest pain	Slight ST elevation inV3 and V4	No	Palliative chemotherapy with cetuximab, carboplatin, and 5-FU	CT Chest	Yes

21	Chua et al. [26]	2017	Tongue	Resection and reconstruction	Right ventricle, minor pericardial effusion	Progressive dyspnea	—	—	Concurrent chemoradiotherapy	Transthoracic echocardiogram	No

22	Cho et al. [15]	2018	Oral Cavity	Induction chemotherapy (doxetaxel/cisplatin/5-FU) followed by concurrent chemoradiotherapy to 66 Gy with cisplatin followed by complete surgical excision	Interventricular septum protruding into right ventricle	Dizziness	Complete AV block	No	Permanent pacemaker placement and palliative chemotherapy	CT Chest and Echocardiogram	Yes

23	Present Study	2018	Left lateral tongue	T-wave inversion in the inferior and V3-V6 leads.	Left ventricle	Chest pressure, dizziness, dyspnea	T-wave inversion	No	Nivolumab	CT Chest	Yes
